# Exploring the Sensory Odor Profile of Sourdough Starter from Ancient Whole-Wheat Flours in the Development of Cookies with Enhanced Quality

**DOI:** 10.3390/foods14040613

**Published:** 2025-02-12

**Authors:** Dubravka Škrobot, Nikola Maravić, Miroslav Hadnađev, Tamara Dapčević-Hadnađev, Mladenka Pestorić, Jelena Tomić

**Affiliations:** Institute of Food Technology, University of Novi Sad, Bul. Cara Lazara 1, 21000 Novi Sad, Serbia; dubravka.skrobot@fins.uns.ac.rs (D.Š.); nikola.maravic@fins.uns.ac.rs (N.M.); tamara.dapcevic@fins.uns.ac.rs (T.D.-H.); mladenka.pestoric@fins.uns.ac.rs (M.P.); jelena.tomic@fins.uns.ac.rs (J.T.)

**Keywords:** ancient wheat sourdough, sensory analysis, Temporal Dominance of Sensations (TDS), Check-All-That-Apply (CATA), Rate-All-That-Apply (RATA)

## Abstract

This study investigates the benefits of sourdough fermentation using ancient whole-wheat flours in the development of cookies, leveraging innovative rapid sensory evaluation methods to highlight their unique sensory attributes and potential health advantages. The spontaneous fermentation of wholegrain wheat, spelt, Khorasan, and emmer flour–water mixtures was monitored, focusing on odor development. Temporal Dominance of Sensations (TDS) was employed to track how sourdough odor unfolds over time while Check-All-That-Apply (CATA) and Hedonic tests were applied to capture the sensory characteristics of sourdough starter samples and consumer overall liking in order to identify sourdough with the most appealing odor for cookie preparation. Based on the result, spelt and Khorasan lyophilized sourdough were used for cookie preparation. Further, Rate-All-That-Apply (RATA) was applied to investigate the sensory profiles of the developed cookies and panelists’ hedonic perceptions and attitudes toward them. The resulting sourdough cookies exhibited higher fiber and comparable protein and fat content, lower energy value with sensory properties comparable to those of commercial samples. This research not only presents a comprehensive selection of sensory methodologies ideal for product development but also offers valuable insights into the sensory profile of sourdough-containing cookies, paving the way for enhanced formulation and strategic commercialization.

## 1. Introduction

In recent years, the modern lifestyle has induced people to adopt healthier eating habits, with a particular focus on the nutritional quality of the foods they choose [1,2]. Incorporating nutrient-dense foods into the diet can provide a diverse array of benefits ranging from improved immune function to enhanced mental and physical performance [3]. These shifting preferences have driven both scientific researchers and the food industry to focus on developing health-promoting products by utilizing more natural ingredients and sustainable processes. In this context, ancient wheat varieties have gained renewed interest, driven largely by the growing demand for traditional products and their potentially superior nutritional profiles compared to modern wheat. These ancient varieties are often praised for their higher fiber content and antioxidant properties [4]. Whole-wheat flour derived from these ancient grains, such as spelt, Khorasan, or emmer, offers unique advantages over conventional wheat flours, although its sensory characteristics—such as taste, aroma, and texture—must be carefully considered to ensure broad consumer acceptance. Specifically, he higher fiber content significantly impacts the rheological behaviour of the dough. The increased fiber can lead to firmer, less extensible dough, making it more challenging to handle and shape. This change in dough consistency can also influence its sensory properties, which may differ from the expectations of consumers accustomed to products made with modern wheat varieties [5]. Additionally, the inherent sensory limitations of whole-wheat flour, such as bitter notes and coarse texture, can further hinder its broader appeal in bakery products. To address these challenges, sourdough fermentation, an ancient biotechnological process, has been recognized as a promising technique for enhancing the sensory and functional qualities of whole-wheat flours by modifying their biochemical and aromatic profiles [6,7]. This process has experienced a resurgence in popularity, largely due to its association with natural, additive-free products and its ability to elevate flavor, texture, shelf life, and the health-promoting properties of baked goods [8,9,10].

Sourdough fermentation relies on the symbiotic interaction between a mixture of lactic acid bacteria and yeasts, driving a series of biochemical transformations that fundamentally alter the dough’s composition and enhance the quality of the final product in numerous ways. During this process, starches are broken down into simple sugars, which are metabolized to produce carbon dioxide for leavening and organic acids that enhance flavor, reduce pH, and extend shelf life. Proteins are partially degraded, improving digestibility and texture, while increased phytase activity from the microflora enhances mineral absorption. These transformations result in sourdough bread with a tangy flavor, complex aroma, better nutrition, and a softer texture [11,12].

In sourdough fermentation, the competition and collaboration between lactic acid bacteria (LAB) and wild yeasts play a pivotal role in shaping the dough’s biochemical and sensory profile. This dynamic interaction, particularly under varying fermentation conditions, determines the type and intensity of organic acids, alcohols, esters, and ketones produced. These volatile compounds are central to the distinctive taste and aroma of sourdough products and are influenced by factors such as the starter culture, the type and quality of flour, fermentation temperature, and dough yield [10]. The maturity of a sourdough starter is a key factor that determines its effectiveness in leavening and flavor production. Bakers typically evaluate a starter’s readiness by sensory attributes such as appearance, flavor, and odor but the scientific description of sensory properties of starter maturity remains limited [13]. In practice, a starter that is too young lacks complex flavors and robust leavening power, while an over-mature starter can become overly acidic and produce fewer bubbles, leading to decreased leavening capacity [14]. The interplay between bacterial organic acid production and yeast-mediated CO_2_ production is complex, with low fermentation temperatures favoring the production of acetic acid, which enhances the tangy flavor of sourdough [15,16]. Therefore, maintaining a controlled acidity level during sourdough fermentation is crucial. This balance is necessary to avoid issues like excessive acidity, which can result in an overly sour or pungent taste while ensuring the proper transformation of substrate components to deliver all the associated benefits [17]. However, the balance between lactic acid and acetic acid in sourdough is not always straightforward, and bakers often struggle to discern the dominance of these acids in their products [18].

On these bases, the present work was planned with the aim of exploiting advanced sensory evaluation methods like Temporal Dominance of Sensations (TDS), Quantitative Descriptive Analysis (QDA), Check-All-That-Apply (CATA), and the Hedonic Test to characterize sourdough odor obtained through the process of spontaneous fermentation of wholegrain flours from ancient wheats and to identify those with the most appealing odor, which would be further used for the development of cookies with distinctive sensory attributes and improved nutritional composition. Additionally, the developed cookies were benchmarked with two commercial cookies of the same type in terms of nutritional, physical (color and texture), and sensory profiles. Hedonic and attitude perceptions toward all the cookies were analyzed by applying the Rate-All-That-Apply (RATA) method.

## 2. Materials and Methods

### 2.1. Materials

The ancient wheat varieties—emmer (*Triticum turgidum* L. subsp. *Dicoccum*), spelt (*Triticum aestivum* L. subsp. *Spelta*), and Khorasan (*Triticum turgidum* L. subsp. *turanicum*)—were sourced directly from a local producer (Poljoprivredno gazdinstvo Spelta Jevtić in Bačko Gradište, Serbia), while a modern wheat variety was supplied by a local milling company (Danubius d.o.o. in Novi Sad, Serbia). All grains were harvested in the same year and dehulled using a large-scale friction de-huller from Heger (Herrenberg, Germany). To produce whole-wheat flour from the modern wheat variety, a Bühler laboratory mill (Uzwil, Switzerland) was used, while the ancient varieties were milled using equipment from Osttiroler Getreidemühlen (Dölsach, Austria). Vegetable fat, sugar, sodium bicarbonate, and ammonium bicarbonate for cookie production were procured from local suppliers.

### 2.2. Sourdough Preparation

Spontaneous fermentation of ancient and modern wheat wholegrain flour was performed in a laboratory incubator (Friocell 111, MMM Medcenter Einrichtungen GmbH, München, Germany) following a back-slopping procedure (every 24 h for 5 days). Once the mature sourdough was obtained, one portion was frozen and lyophilized, while the rest was regularly refreshed and used fresh in the cookie formulations. The detailed procedure of sourdough preparation and lyophilization was described in our previous papers Tomić et al. [19] and Maravić et al. [20]. Briefly, flour samples and demineralized water were combined in a 1:1 (*w*/*w*) ratio, resulting in dough with a yield of 200%. The dough was incubated at 25 °C for 24 h for initial fermentation. Subsequently, four back-slopping steps were performed, mixing fermented dough with flour and water in a 1:2:2 (*w*/*w*) ratio at 24 h intervals, leading to the establishment of mature sourdough characterized by stable microflora. Following maturation, the refreshment process was conducted weekly, combining mature dough with fresh flour and demineralized water in a 1:2:2 ratio, and fermenting at 25 °C for 6 h. This duration was optimized according to findings by Tomić et al. [19] to achieve the desired pH and maturation stage. One portion of mature sourdough was lyophilized after a 6 h refreshment fermentation, using a freeze dryer under vacuum (0.128 mbar) at 20 °C for 36 h, following initial freezing at −80 °C for 24 h. The lyophilized samples were stored in sealed bags at room temperature for subsequent analysis, while the remaining sourdough was kept fresh for use in cookie formulations.

### 2.3. Sourdough Starter Sensory Analysis

#### 2.3.1. Dynamic Odor Perception via Temporal Dominance of Sensations (TDS)

Twenty sensory assessors (15 women and 5 men, aged between 25 and 45 years) with at least one year’s experience in quantitative descriptive analysis of various food products, but without previous practical experience in TDS took part in this study. Therefore, three 60 min training sessions were oriented to explain the concept of dominant sensations and to identify the odor attributes that trigger assessors’ attention the most at a given time. Training sessions were performed with different sourdough starters in different maturity stages, and assessors agreed on the list of 10 odor attributes that were the most frequently perceived (yogurt, sour milk, yeast, cheese, dough, fruity, acetic acid, brans, flour, lactic acid). These attributes were presented simultaneously as buttons on a computer screen, together with “Start” and “End” buttons. The attribute order on the screen was kept constant for each assessor during the evaluation of all samples; however, this order was randomized across assessors to reduce potential bias [21]. During training sessions and just before the actual test session, assessors were shown a demonstration video explaining the evaluation procedure to familiarize them with the methodology and how to use the program.

For TDS measures, samples of sourdough starters were prepared in such a way that, respecting the required fermentation time, all samples (7 in total per wheat culture) were ready for analysis at the same time. Evaluation of all samples per wheat type was performed within one day and was replicated during three successive days. Samples were delivered to the assessors in a glass jar with a lid coded with 3-digit random numbers. The assessors were instructed that once they removed the lid, they had to click on the “Start” button on the screen to begin the evaluation. During an evaluation, mixing a sample carefully was allowed to release all volatiles, and assessors had to select the odor attribute that they perceived as the most dominant. When assessors noticed that one dominant odor perception changed, they had to score a new dominant sensation, until perception ended, when they had to click the “End” button. The assessors were free to select as many attributes as they wanted during the evaluation of the samples, including re-selecting an attribute more than once during the test.

#### 2.3.2. Quantitative Descriptive Analysis (QDA)

Sensory profiling of sourdough odor was performed only on mature sourdough samples (those collected after six hours of activation period) prepared from different wheat cultivars by using the same odor attributes that were analyzed during the TDS test. The intensities of perceived odor attribute assessors evaluated on continuous 10 cm unstructured line scales.

All sensory analyses were performed in the sensory laboratory of the Institute of Food Technology (University of Novi Sad) in individual booths equipped with a computer. Each assessor evaluated all samples, and the order of sample presentation was randomized across them. Between samples, assessors had a 15 min break to rest their sense of odor.

### 2.4. Consumer Study of Sourdough: Overall Liking Study and Check-All-That-Apply (CATA)

A total of 48 participants (30 men and 18 women, aged 37–54 years), professional bread makers and participants in a Sourdough open day organized by the Institute of Food Technology in Novi Sad, were included in the study. Activated sourdough starter samples, placed in lidded glass jars marked with three-digit numbers, were distributed in a randomized order among participants. The participants were instructed to sniff the sample as soon as they opened a jar, then to stir the sample with a plastic spoon and to sniff the sample again. At the beginning, the participants were asked to evaluate how much they liked the odor of each sourdough sample and to mark whether this odor was pleasant or not for them. The evaluation was performed on a 9-point hedonic scale (1 = dislike extremely; 5 = neither like nor dislike; 9 = like extremely). The liking study was followed by CATA questions that included 18 terms: alcoholic, yeasty, beer, red wine, lactic acid, sour dairy, matured hard cheese, fruity, sharp acidic, porridge, grain field, bread, soil, brans, flour, putrid off-notes, toasted bread, soy sauce. The participants were instructed to choose all the attributes that apply to each analyzed sourdough sample. The order of terms was presented randomly between the products and across the assessors.

The study was approved by the Ethics Committee of the Institute of Food Technology in Novi Sad, University of Novi Sad, Serbia (Ref. No. 175/I/7-3).

### 2.5. Cookies Preparation

Cookie samples were prepared exclusively using the type of wheat flour for which sourdough was selected based on its pleasant odor characteristics, as determined by the TDS method. Four cookie formulations were prepared under identical processing conditions by replacing a portion of flour with lyophilized sourdough derived from the same wheat variety. Ingredients used for cookie preparation together with their formulations are presented in Table 1.

Cookie dough preparation started with mixing vegetable fat with sugar for 2 min followed by the addition of water to create a homogeneous mixture. All other ingredients were then added and mixed for an additional 3 min. The resulting dough was sheeted to a thickness of 3.0 mm using a pilot-scale dough sheeter (Macpan, Thiene, Italy). Circular cookies (diameter 45 mm) were cut and baked at 190 °C for 11 min in a laboratory oven (MIWE gusto^®^ CS, Arnstein, Germany). After cooling at room temperature for 1 h, the cookies were sealed in polyethylene bags for further analysis.

### 2.6. Cookies Analysis

The developed cookies were characterized in terms of nutritional (proximate composition profile), color, and textural and sensory properties (descriptive and hedonic profile) and benchmarked against the two existing products of the same type.

#### 2.6.1. Cookie Proximate Composition

AOAC standard methods were employed for the determination of protein (method 920.87), fat (method 922.06), ash (method No. 923.03), and moisture mass fractions (method No. 925.09) content in produced cookie samples. Megazyme Total Dietary Fiber Assay Kit (Neogen, Lansing, MI, USA) was used for the analysis of total dietary fiber content of the produced cookies following methods 985.29 (AOAC) and 32-07 (AACC) [22]. Available carbohydrates were calculated by subtracting the total mass of water, protein, fat, ash, and dietary fiber (in grams per 100 g of the sample). The energy value was assessed in accordance with European Regulation No. 1169/2011 [23].

#### 2.6.2. Colour Determination of Cookies

Cookie samples (n = 5) were taken randomly from the batch, 24 h after baking, and served for measurement of the color properties. Measurements were performed on a cookie’s top surface in five points using a Minolta CR-400 (Konica Minolta Co., Osaka, Japan) Chroma meter. The results were expressed in terms of L*—lightness (from 0 (black) to 100 (white)), a*: greenness/redness (from a* < 0 (green) to a* > 0 (red)), b*: blueness/yellowness (from b* < 0 (blue) to b* > (yellow)). Browning index (BI) was calculated from Equation (1) [24]:

(1) $$\mathrm{BI}=\frac{\left[100\;\times \;\left(X-0.31\right)\right]}{0.17}\;\mathrm{where}\;X={(a}^{*}+1.75L^{*})/(5.645L^{*}+a^{*}-0.3012b^{*})$$

Yellowness Index was calculated from Equation (2) [25]:

(2) $$\mathrm{Yellowness}\;\mathrm{Index}=\;142.86\;\frac{b^{*}}{L^{*}}$$

#### 2.6.3. Textural Properties of Cookies

The texture properties of cookies were assessed by measuring hardness and fracturability using the TA-XTPlus Texture Analyser (Stable Micro Systems, Godalming, UK), equipped with a three-point bend rig (HDP/3PB) and operated via Exponent software (v8.10). Measurements were conducted in distance mode, following the test setup outlined by Maravić et al. [20]. In brief, the texture analysis was conducted using a three-point bend rig with two adjustable supports positioned 30 mm apart. The upper blade was aligned equidistantly between the supports and initially descended at a speed of 1 mm/s until a contact force of 50 g was detected. Once triggered, the crosshead traveled 5 mm into the cookie at a speed of 3 mm/s. Six samples were randomly selected from each cookie type 24 h post-baking to ensure consistent results.

#### 2.6.4. Sensory Analysis of Cookies

The sensory profile of cookies was determined by applying the Rate-All-That-Apply (RATA) method according to which panelists had to select terms that were relevant to the analyzed samples from a list of attributes and then rate the perceived intensity. Sensory attributes were categorized into appearance (shape unevenness, surface unevenness, yellow color, brown color, color unevenness, pore unevenness), odor (sweet, sour, yeast, grains, flour), taste (sweet, sour, bitter), flavor (sweet, sour, whole wheat, vegetable fat/oil, overall flavor intensity, astringency, persistence, aftertaste), and textural properties (texture evaluated manually: crumbliness and hardness; texture evaluated during initial bite: hardness, fracturability, crunchiness; texture evaluated during chewing: hardness, fracturability, chewiness, tooth pack, dryness, oily mouth, coating, granular, chalky), which is explained in detail in our previous paper Maravić et al. [20]. The RATA questions also included the following hedonic and attitude terms: overall liking, healthy, enjoyable, satisfying, and unpleasant. The RATA questions were presented together with a 5-point rating scale where 0 indicates that attribute is not present, 1 indicates that attribute is very weakly noticeable and 5 indicates that attribute is extremely noticeable. All 15 trained sensory assessors, comprising 10 females and 5 males aged 25 to 50, evaluated the cookie samples. The samples were distributed in a randomized order and labeled with three-digit numbers for identification. Mineral water was used for palate cleansing between samples.

The sensory analysis was performed at the sensory laboratory at the Institute of Food Technology in Novi Sad. The study was approved by the Ethics Committee of the Institute of Food Technology in Novi Sad, University of Novi Sad, Serbia (Ref. No. 24-52-3).

### 2.7. Data Analysis

Results are presented as mean ± standard deviation from replicated analyses. Analysis of variance (ANOVA) followed by Tukey’s Honest Significant Difference test (*p* < 0.05) was employed to assess the significance of differences between sample means. Cochran’s Q test was carried out on the CATA and RATA results in order to identify significant differences between samples for each of the attributes.

All statistical analyses were conducted using the XLSTAT software package version 2023.3.1 (Addinsoft, New York, NY, USA).

## 3. Results and Discussion

### 3.1. Sourdough Starter Sensory Analysis

#### 3.1.1. Dynamic Odor Perception via TDS

In order to describe the dynamic changes in the sourdough odor profile during the mature starter activation, the Temporal Dominance of Sensations (TDS) method was performed. Since this method can highlight the dominant odor of sourdough, allowing better identification of the subtle differences between various samples [26], we utilized it to select samples that exhibited the most desirable and pleasant odor characteristics.

**Figure 1:**
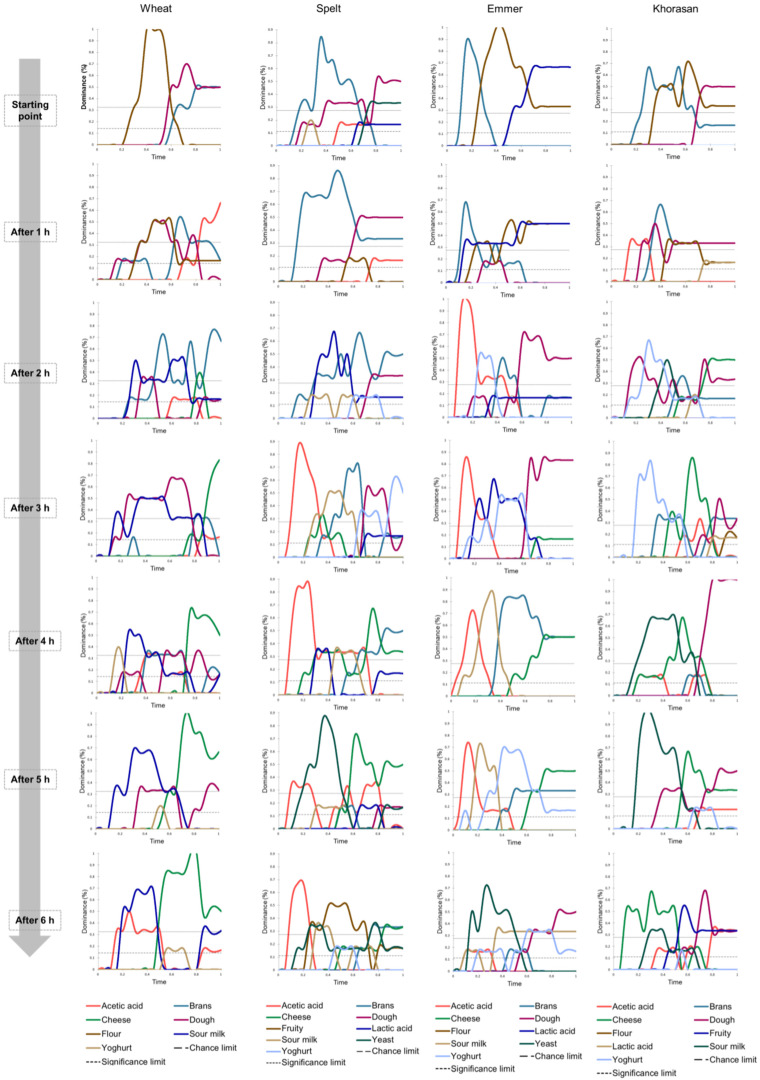
TDS plots for wheat, spelt, emmer, and Khorasan sourdough starter samples over a six-hour period of activation.

Figure 1 presents smoothed TDS curves for each evaluated sample. Odor attributes with dominance levels below the chance threshold were not considered significant. The obtained TDS curves revealed clear distinctions in the dominant odor attributes among sourdough starter samples prepared from different wheat cultivars. At the initial stage of sourdough starter activation (“starting point”), the odor profiles of the samples were relatively simple and similar. The dominant odors detected were primarily related to the raw materials: bran odor (common to all samples), flour odor (present in all samples except spelt sourdough), and dough odor (not observed in emmer sourdough). Notably, emmer sourdough exhibited a unique pattern, with lactic acid odor becoming the most dominant from the middle of the evaluation period, corresponding to the period after sample stirring.

Over the 6 h observation period, the odor profiles of the sourdough samples became increasingly complex, further highlighting the differences between the analyzed samples. Between 2 and 6 h, the TDS curves for emmer sourdough showed the presence of acetic acid odor, followed by sour milk, yogurt, and cheese-like odors. By the 6 h mark, yeast odor was the most dominant attribute in active emmer sourdough, aligning with the total yeast and Lactobacillus content (published previously in our paper Tomić et al. [19]). Similarly, yeast odor was detected in spelt sourdough, which exhibited the highest yeast content. An interesting observation was the prevalence of sour milk odor, often accompanied by cheese-like notes, in all samples starting from 4 h of activation and persisting until the end of the period. For the spelt and Khorasan sourdough starters, these fermented milky odor attributes were occasionally interspersed with short periods of increased dominance of fruity odor notes.

#### 3.1.2. Static Descriptive Analysis of Sourdough Odor via QDA

**Figure 2:**
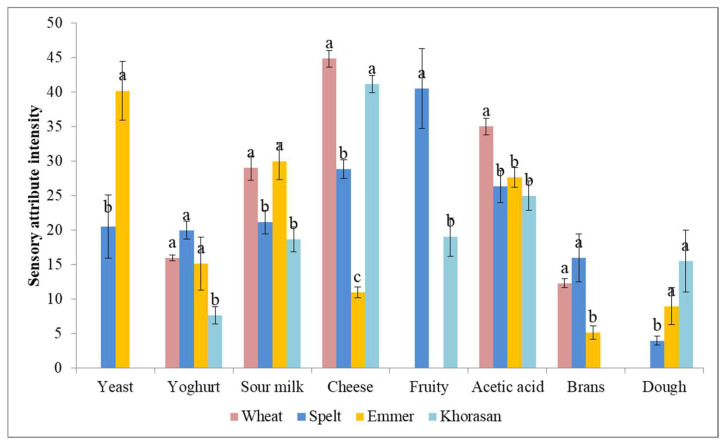
Average intensities of odor attributes in QDA (different letters above the bars indicate statistical differences (*p* < 0.05) among the samples according to Tukey’s minimum square difference test).

In scientific research, starter maturity is typically evaluated by examining the stability of its pH, rise, or microbiota, where specific species of bacteria and yeasts consistently emerge at particular stages (i.e., early or mature). However, bakers traditionally assess a starter’s maturity by evaluating its sensory traits, such as its visual appearance or odors [14]. Quantitative descriptive analysis of mature sourdough starters in the present study was performed for ten odor attributes. Lactic acid and flour odor notes were not perceived in any of the analyzed mature sourdough starters regardless of the wheat cultivar (Figure 2). These findings are in accordance with the baker observations presented by Calvert et al. [14], who perceived flour odor only in young sourdough, but not in the vibrant and mature stages. The odor profile of mature spelt sourdough was the most complex, consisting of eight distinct odor notes, with dominant characteristics reminiscent of fruit and a prominent presence of sharp, fermented dairy undertones. Contrary to this, the odor profile of mature wheat sourdough was simpler, but stronger and sharper, compared to that of the other samples with more acidic dairy-like notes, with cheese notes being the most dominant one. However, although mature Khorasan sourdough possessed similar odor characteristics to wheat sourdough, the presence of fruity and dough notes, and significantly (*p* < 0.05) lower intensities of perceived tangy attributes, gives this sample a more pleasant and more balanced odor profile (according to the Section 3.2). The odor of emmer sourdough was driven by a yeasty odor with the prominent presence of fermented dairy and fermentation by-products. The results obtained align with the conclusions of a previously published study by Calvert et al. [14], which demonstrated that bakers distinguish starter maturity based on sensory characteristics. According to their findings, a young starter lacks a complex aroma and is not an effective leavening agent. A vibrant starter, conversely, is at its peak of fermentation and exhibits sweet and sour odor notes. In contrast, a starter that has passed its peak is considered mature and is characterized by strong, pungent, vinegary, and distinctly fermented odors.

### 3.2. Overall Liking and CATA Questions

**Figure 3:**
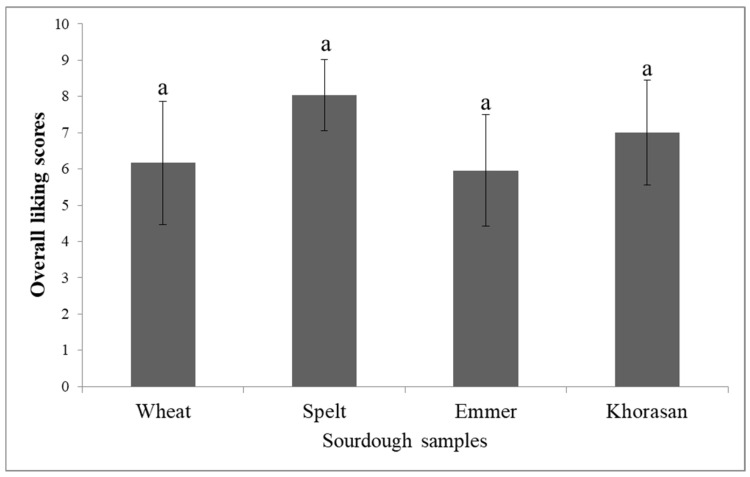
Overall liking of mature sourdough odor (different letters above the bars indicate statistical differences (*p* < 0.05) among the samples according to Tukey’s minimum square difference test).

Figure 3 shows the results for the overall liking of sourdough starters’ odor. The applied analysis of variance did not show significant differences (*p* < 0.05) in overall liking between the odor profiles of the sourdough starters (Figure 3). Although this result suggests that the participants did not like any of the odor samples more than the others, the average scores for the odor of the spelt (8.04) and Khorasan (7.00) sourdough starters were slightly higher than those of the odor of the wheat (6.17) and emmer (5.96) samples.

**Figure 4:**
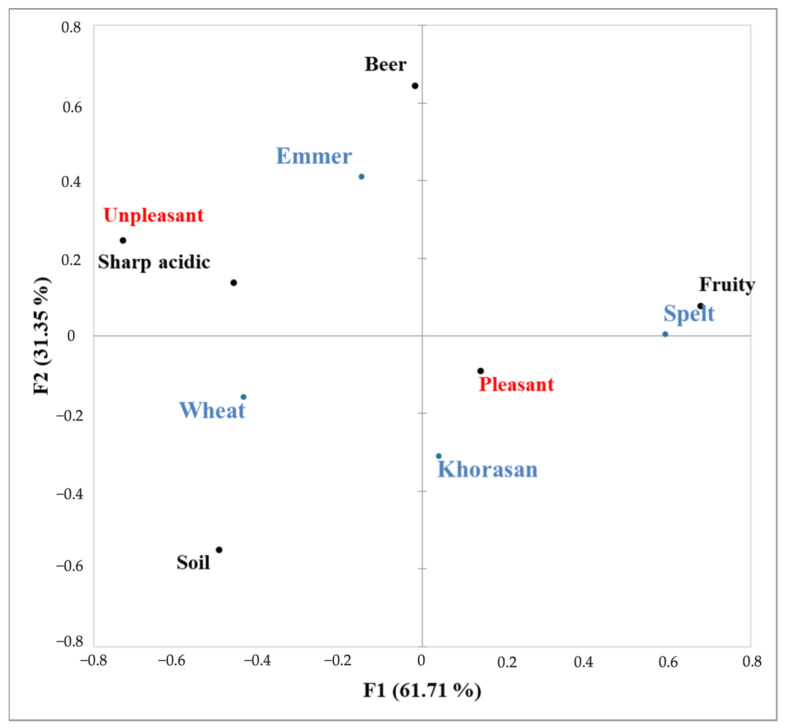
Sensory map obtained using the CATA sensory analysis of sourdough starters` odor profile.

Among the 18 listed terms in the CATA questionnaire, only six presented significant differences between the samples according to the applied Cochran’s Q test. The sensory map obtained throughout the multivariate correspondence analysis is presented in Figure 4. Using two dimensions, 93% of total data variability was explained. Clear separation of samples was observed based on the pleasantness of the odor profiles. The CATA analysis revealed that the pleasantness of the spelt sourdough starter is associated with its fruity aroma, which bakers link to vibrant starters that are likely to produce the desired outcome of a final product [14]. Given that the odor profile of Khorasan sourdough also includes fruity notes, the pleasantness of its sourdough sample can similarly be attributed to this odor characteristic. In contrast, the wheat and emmer samples were considered unpleasant primarily due to the sharp acidic notes, which could be a result of sourdough starters that had passed their peak fermentation stage [14]. Additionally, other factors related to sourdough fermentation process parameters, such as fermentation time, temperature, dough yield, flour ash content, and oxygen levels, may have also contributed to the undesirable odor. Salovaara and Valjakka [27] found that the type of flour was the most significant factor influencing acid production. In bread made with dark flour (ash content 1.64%), the acetic acid concentration was nearly twice as high as that in bread made with white flour (ash content 0.86%), while the lactic acid level was 30 to 50% greater. The pronounced acidic odor of wheat sourdough was reported by Kati et al. [28] who considered that such a sensation generally is not appreciated by consumers in many countries.

### 3.3. Cookie Analysis

Thanks to the applied different sensory techniques (TDS, Overall Liking, and CATA) that underscore the critical role of sensory properties in choosing the most suitable starters, two sourdough starters—spelt and Khorasan—were selected for cookie preparation.

#### 3.3.1. Proximate Composition of Cookies

**Figure 5:**
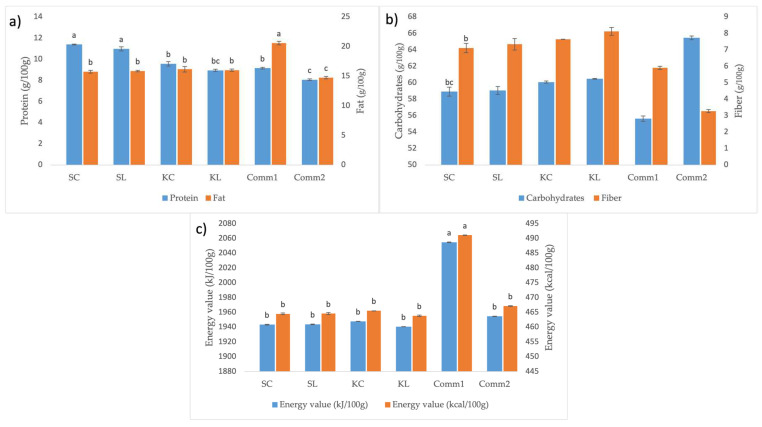
Proximate composition of cookies: (**a**) protein and fat content, (**b**) carbohydrate and fiber content, (**c**) energy value (different letters above the bars indicate statistical differences (*p* < 0.05) among the samples according to Tukey’s minimum square difference test).

The chemical composition of cookies developed in this study is presented in Figure 5 along with the information about the chemical composition of commercially available cookies that were chosen based on whole-wheat flour and similar composition of other ingredients. The chemical composition of commercial samples was obtained from their nutrition labeling declarations. The cookies developed in this study, as well as Comm1, had significantly higher protein content than Comm2 (Figure 5a). The highest protein content was measured in the spelt cookies, although it was slightly lower in counterparts containing lyophilized sourdough. This finding is supported by our previous work, where it was shown that spelt flour is richer in proteins than Khorasan and wheat flour [20,29]. The fat content of the developed cookies was significantly (*p* < 0.05) lower than in Comm1 but higher than in Comm2. As a result, the developed cookies had a significantly (*p* < 0.05) reduced energy value compared to Comm1. Conversely, all the developed cookies had significantly (*p* < 0.05) higher fiber content compared to both commercial samples (Figure 5b). Furthermore, all the developed cookies can be labeled as ‘high in fiber’ as they contain more than 6 g of fiber per 100 g, according to Regulation (EC) No 1924/2006 [30]. The carbohydrate content of the developed samples was intermediate between the two commercial samples, with those containing lyophilized sourdough having a higher carbohydrate content compared to the counterparts without it.

#### 3.3.2. Color Measurement

**Figure 6:**
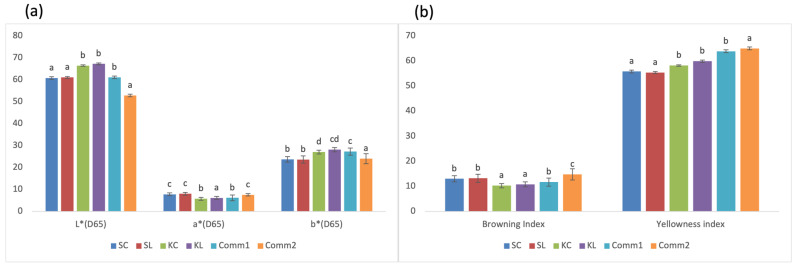
Color properties of cookie samples: (**a**) lightness (L*), redness (a*), yellowness (b*); (**b**) browning index, yellowness index (different letters above the bars indicate statistical differences (*p* < 0.05) among the samples according to Tukey’s minimum square difference test).

The color of a product is one of the most important properties of its appearance that can have a significant impact on consumer acceptance and purchasing intentions [31]. Color measurements showed significant (*p* < 0.05) differences between samples for all analyzed parameters (Figure 6). Regarding the cookies’ lightness (L*), there were no observed significant (*p* < 0.05) differences between cookie samples prepared from the same flour, indicating that flour type has a higher impact on this parameter than the sourdough addition. The same was observed for redness (a*) of all samples and yellowness (b*) of both spelt cookies. However, the addition of lyophilized Khorasan sourdough increased the yellowness of the cookies. The highest lightness was observed in all samples containing Khorasan, while the Comm2 sample was the darkest and with significantly (*p* < 0.05) higher browning and yellowness index. Both spelt cookie samples were more similar to the Comm2 sample in terms of a* and b*, while the redness of the Khorasan cookies was comparable to the Comm1 sample.

#### 3.3.3. Textural Properties of Cookies

**Figure 7:**
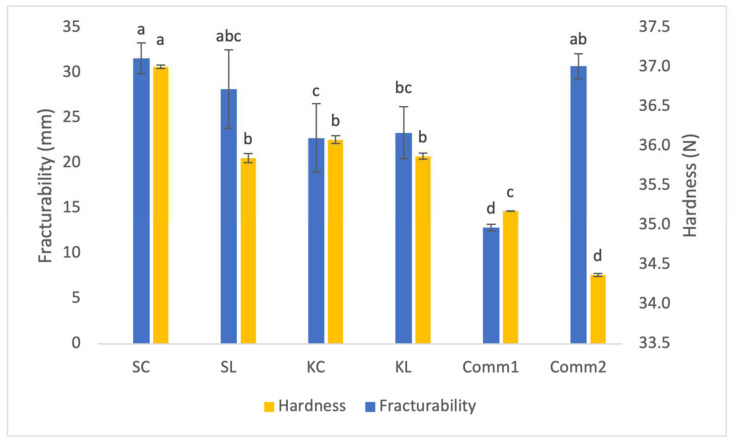
Textural properties of cookies (different letters above the bars indicate statistical differences (*p* < 0.05) among the samples according to Tukey’s minimum square difference test).

Figure 7 illustrates the results of an analysis of the cookies’ textural properties. The hardness of the cookies refers to how easily they can be broken by compression [32]. The addition of lyophilized sourdough influenced significantly only the hardness of the spelt sourdough cookies. Both commercial samples were significantly less hard, whereas the control spelt cookie showed significantly (*p* < 0.05) higher hardness. This may be due to the higher content of proteins in the produced cookies, which contributes to the hardness increase [33]. Alternatively, the higher hardness of these cookies may be a result of the significantly lower fat content. Fat reduction in cookie formulations typically leads to increased hardness, as fat plays a crucial role in lubricating and coating the matrix. With less fat, flour and fiber particles have greater access to water [34], which enhances hydration and results in the formation of harder doughs, and consequently, harder cookies [35]. Additionally, doughs containing full fat incorporate air more effectively than their fat-reduced counterparts [36], further contributing to increased cookie hardness. The obtained results for cookie fracturability showed that the addition of lyophilized sourdough into the cookie formulation slightly decreased these values; however, the observed changes were not significant (*p* < 0.05).

#### 3.3.4. Sensory Analysis of Cookies

**Figure 8:**
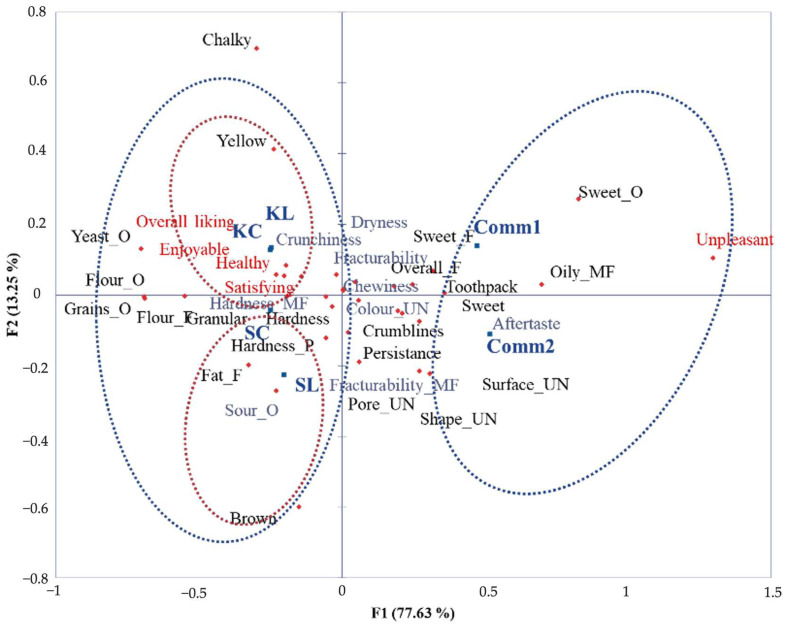
Graphical representation of products and attributes obtained by applying RATA sensory analysis (SC: spelt control cookies; SL: spelt cookies with lyophilized sourdough; KC: Khorasan control cookies; KL: Khorasan cookies with lyophilized sourdough; Comm1 and Comm2: commercial samples. Sensory properties presented in gray are not statistically significant according to ANOVA (*p* > 0.05)).

The sensory map obtained through the application of Rate-All-That-Apply (RATA) sensory analysis is presented in Figure 8. This is a rapid sensory profiling method and variation of the Check-All-That-Apply method in which participants choose relevant terms from a predefined list and then assess the intensity or applicability of each term for the focal sample [37]. The obtained two-dimensional map explains more than 90% of data variability. The performed ANOVA (Supplementary Table S1) showed that for all analyzed attributes except colour_UN, sour_O, aftertaste, fracturability, crunchiness, hardness_MF, chewiness, fracturability_MF, and dryness, the product variable is significant at the 5% threshold, meaning that these attributes discriminate among samples. Samples were separated into two groups mostly based on panelists’ attitudes and hedonic perceptions of the cookies. Considering sensory properties, the chart shows opposition between odor (sweet odor on one side, yeast, flour, and grains odor on the other) and color properties (yellow and brown). The first group consists of samples developed within our study (SC, SL, KC, and KL), and these samples are considered more healthy, enjoyable, satisfying, and pleasant, with a higher overall liking. This group can be divided by wheat variety into two subgroups. One group is formed by Khorasan cookie samples (KC and KL) described as more yellow and with the highest overall liking. Within the Khorasan cookies, there were no noticeable differences in terms of all analyzed sensory and hedonic properties, meaning that sourdough addition did not deteriorate sensory properties. Conversely, within the second subgroup that was made up of spelt cookie samples (SC and SL), described as more brown, hard, and granular with a fat flavor, the addition of lyophilized sourdough slightly decreased the overall liking score. The second group was formed of commercial samples that were generally considered unpleasant, with a noticeable sweet taste, odor, and flavor, and a crumbly texture and oily mouthfeel.

## 4. Conclusions

This study provides valuable insights into the dynamic and static sensory odor profiles of ancient wheat sourdough starters, contributing to a deeper understanding of their role in product development. The results support the following key findings:

- Advanced sensory analysis techniques, including Temporal Dominance of Sensations (TDS), Check-All-That-Apply (CATA), and Quantitative Descriptive Analysis (QDA), proved to be effective tools in identifying the most appealing sourdough starters.
- According to these techniques, spelt and Khorasan sourdoughs were selected as the most promising candidates for cookie formulation.
- Sourdough-enriched cookies exhibited comparable nutritional, color, and textural properties to commercial cookies, with a notable increase in fiber content and enhanced sensory appeal.
- Khorasan cookies with lyophilized sourdough starter were considered the most enjoyable, satisfying, and health-promoting option.

This research not only highlights the utility of advanced sensory methods for product development but also offers significant insights into the sensory profiles of sourdough-enriched cookies. These findings open the door for refined formulations and the strategic commercialization of innovative, health-conscious cookie products. The application of these sensory techniques effectively distinguishes between commercial and newly developed cookies with similar sensory properties, offering practical strategies for further innovation within this growing product category.

## Figures and Tables

**Table 1:** Ingredients used for cookie formulations.

Ingredients (g/100 g)	Spelt_C	Spelt_L	Khorasan_C	Khorasan_L
Flour (g)	100	75	100	75
Sourdough lyophilized (g)	-	25	-	25
Vegetable fat	20	20	20	20
Sugar	15	15	15	15
NaCl	0.5	0.5	0.5	0.5
NaHCO_3_	0.3	0.3	0.3	0.3
NH_4_HCO_3_	0.2	0.2	0.2	0.2
Water	29.1	31.3	28.2	30.7

## Data Availability

The original contributions presented in this study are included in the article; further inquiries can be directed to the corresponding author.

## Supplementary Materials

The following supporting information can be downloaded at: https://www.mdpi.com/article/10.3390/foods14040613/s1, Table S1: ANOVA summaries for Rate-All-That-Apply results.
