# Supplementary material for: Exploring the Sensory Odor Profile of Sourdough Starter from Ancient Whole-Wheat Flours in the Development of Cookies with Enhanced Quality
Source: Foods. 2025 Feb 12;14(4):613. doi: 10.3390/foods14040613 (PMC11854415; doi:10.3390/foods14040613)
Supplement: Supplementary file 1 [file foods-14-00613-s001.zip › foods-3418177-supplementary.pdf]

**Supplementary Table S1.** ANOVA summaries for Rate-All-That-Apply results

| | $R^2$ | F | Pr > F | Sample | |
|---|---|---|---|---|---|
| | | | | F | Pr > F |
| Shape_UN | 0.621066 | 10.33821 | <0.0001 | 12.63576 | <0.0001 |
| Surface_UN | 0.566046 | 8.227686 | <0.0001 | 13.19198 | <0.0001 |
| Yellow | 0.385513 | 3.95728 | <0.0001 | 3.660173 | 0.005 |
| Brown | 0.498545 | 6.271091 | <0.0001 | 9.603675 | <0.0001 |
| **Colour_UN** | **0.559491** | **8.011422** | **<0.0001** | **1.655322** | **0.155** |
| Hardness_P | 0.471514 | 5.627705 | <0.0001 | 7.411992 | <0.0001 |
| Crumblines | 0.483045 | 5.893933 | <0.0001 | 6.718043 | <0.0001 |
| Pore_UN | 0.519228 | 6.81224 | <0.0001 | 7.919208 | <0.0001 |
| Sweet_O | 0.632622 | 10.86178 | <0.0001 | 22.41047 | <0.0001 |
| **Sour_O** | **0.338471** | **3.227324** | **0.001** | **1.955009** | **0.094** |
| Yeast_O | 0.602186 | 9.548188 | <0.0001 | 10.61161 | <0.0001 |
| Grains_O | 0.612695 | 9.978406 | <0.0001 | 7.38871 | <0.0001 |
| Flour_O | 0.836987 | 32.38662 | <0.0001 | 75.19593 | <0.0001 |
| Sweet | 0.619891 | 10.28676 | <0.0001 | 18.87452 | <0.0001 |
| Sweet_F | 0.513769 | 6.664924 | <0.0001 | 13.79051 | <0.0001 |
| Flour_F | 0.715931 | 15.8971 | <0.0001 | 35.12013 | <0.0001 |
| Fat_F | 0.608057 | 9.785696 | <0.0001 | 2.988496 | 0.016 |
| Overall_F | 0.409802 | 4.379723 | <0.0001 | 4.317314 | 0.002 |
| Persistance | 0.576633 | 8.591189 | <0.0001 | 6.881187 | <0.0001 |
| **Aftertaste** | **0.573743** | **8.490154** | **<0.0001** | **1.8368** | **0.115** |
| **Fracturability** | **0.519477** | **6.81903** | **<0.0001** | **0.325397** | **0.896** |
| Hardness | 0.500188 | 6.312435 | <0.0001 | 3.982857 | 0.003 |
| **Crunchiness** | **0.700611** | **14.76083** | **<0.0001** | **1.977445** | **0.091** |
| Granular | 0.563532 | 8.14399 | <0.0001 | 5.555326 | 0.000 |
| Toothpack | 0.423641 | 4.636347 | <0.0001 | 3.796511 | 0.004 |
| **Hardenss_MF** | **0.648023** | **11.61306** | **<0.0001** | **2.244028** | **0.058** |

| | $R^2$ | F | Pr > F | Sample | |
|---|---|---|---|---|---|
| | | | | F | Pr > F |
| **Chewiness** | **0.871418** | **42.74826** | **<0.0001** | **0.241621** | **0.943** |
| **Fracturability_MF** | **0.63066** | **10.77057** | **<0.0001** | **1.604051** | **0.168** |
| **Dryness** | **0.171886** | **1.309245** | **0.225** | **0.327287** | **0.895** |
| Oily_MF | 0.367562 | 3.665917 | 0.000 | 4.246656 | 0.002 |
| Chalky | 0.28541 | 2.519322 | 0.006 | 2.889524 | 0.019 |
| Overall liking | 0.643298 | 11.37569 | <0.0001 | 28.31457 | <0.0001 |
| Healthy | 0.630901 | 10.78175 | <0.0001 | 24.9814 | <0.0001 |
| Enjoyable | 0.723967 | 16.54357 | <0.0001 | 37.83318 | <0.0001 |
| Satisfying | 0.719571 | 16.18531 | <0.0001 | 33.68707 | <0.0001 |
| Unpleasant | 0.924006 | 76.69446 | <0.0001 | 197.0452 | <0.0001 |

Sensory properties in bold are those for which Product variable is not significant at the 5% threshold.
